# A year in pharmacology: new drugs approved by the US Food and Drug Administration in 2024

**DOI:** 10.1007/s00210-025-04020-2

**Published:** 2025-03-31

**Authors:** Zinnet Sevval Aksoyalp, Gizem Kayki-Mutlu, Leszek Wojnowski, Martin C. Michel

**Affiliations:** 1 Department of Pharmacology, Faculty of Pharmacy, Izmir Katip Celebi University, Izmir, Turkey; 2 Department of Pharmacology, Faculty of Pharmacy, Ankara University, Ankara, Turkey; 3 Department of Pharmacology, University Medical Center, Johannes Gutenberg University, Mainz, Germany

**Keywords:** FDA, New drugs, First-in-class, Next-in-class, Cell-based therapy, Rare diseases, Gene therapy, Monoclonal antibody, Orphan drugs

## Abstract

The US Food and Drug Administration approved 50 new drugs and nine new cellular and gene therapy products in 2024, i.e., a total of 59 new medical therapies. The latter group represented three treatments each for oncology and hematology/immunotherapy, and one each for neurology, genetic disorders, and cardiovascular disorders. Oncology, hematology/immunotherapy, and neurological disorders (14, six, and seven, respectively) also were highly prevalent among classic medications. Looking at trends over the past 5 years, we observe a greater share in first-in-class medications, more fast-track approvals, and mRNA/gene/cell-based therapies. While small molecules remain the largest fraction, their percentage has been declining substantially over the past 5 years. Taking together, these findings testify to the commitment of the pharmaceutical industry for innovative treatments, including conditions for which no approved therapies existed. On the other hand, there also is a trend for approvals for narrowly focused conditions such as tumors defined by genetic alterations.

## Introduction

Patterns of new drug approvals can provide insight into the activities and priorities of the pharmaceutical industry and into trends in novel therapeutic approaches. Following an analysis of approvals by the US Food and Drug Administration (FDA) for 2020–2023 (Kayki-Mutlu and Michel 2021, Kayki-Mutlu et al. 2022, Kayki-Mutlu et al. 2023, Kayki-Mutlu et al. 2024), we now provide an analysis of such approvals in 2024. Except for a dip associated with the COVID-19 pandemic in 2022, there is a stable flow of about 50 newly approved drugs per year.

As in our previous annual reviews, we briefly summarize key efficacy and tolerability data for each newly approved drug. We classify the degree of innovation as first-in-indication, i.e., drugs for the treatment of a condition for which no approved medical treatments existed; first-in-class, i.e., drugs with a molecular mechanism of action that had not been used by previously approved medical treatments; and next-in-class, i.e., novel chemical or biological entities that exploit a molecular mechanism already available for the treatment of the same condition (Table 1). Table 2 breaks down the approvals according to the molecular structure (small molecules, antibodies, peptides and proteins, and cellular and gene therapy); we have not grouped antibodies with peptide and proteins (technically they are also proteins) because their role as therapeutics differs considerably from other therapeutic proteins. The increasingly common orphan status is given in Table 3. All approvals are discussed according to therapeutic areas. Given that this is the fifth in a series of annual reviews, we for the first time also discuss trends over the past 5 years for the above parameters.

**Table 1:** Newly approved drugs grouped by novelty. For definitions, see the “Introduction” section. Percentages are those of first-in-indication, first-in-class, and next-in-class drugs with all drugs (included cellular and gene therapies) approved in 2024 taken as 100%. Cellular and gene products are depicted by italics. Where available, the International Nonproprietary Name stems in drug names have been highlighted by underlining based on the WHO Stem Book (https://cdn.who.int/media/docs/default-source/international-nonproprietary-names-(inn)/inn-bio-review-2022.pdf?sfvrsn=f8db166f_3&download=true; https://cdn.who.int/media/docs/default-source/international-nonproprietary-names-(inn)/stembook-2018.pdf)

First-in-indication (*n*=6, 10.2%)	Approved for	First-in-class (*n*=26, 44.1%)	Approved for	Next-in-class (*n*=27, 45.8%)	Approved for
*Acellular tissue engineered vessel-tyod*	Extremity vascular trauma	Aprocitentan	Hypertension	Acoramidis	Cardiomyopathy of wild-type or variant transthyretin-mediated amyloidosis
Arimoclomol	Niemann-Pick disease type C	*Afamitresgene autoleucel*	Unresectable or metastatic synovial sarcoma	Cefepime, enmetazobactam	Complicated urinary tract infections
*Atidarsagene autotemcel*	Metachromatic leukodystrophy	Axatilimab-csfr	Chronic graft-versus-host disease	Ceftobiprole medocaril sodium	Bloodstream infections, bacterial skin and associated tissue infections, and community-acquired bacterial pneumonia
Mavorixafor	WHIM syndrome	Berdazimer	Molluscum contagiosum	Concizumab-mtci	Hemophilia A or B
Olezarsen	Familial chylomicronemia syndrome	Crinecerfont	Classic congenital adrenal hyperplasia	Cosibelimab-ipdl	Metastatic or locally advanced cutaneous squamous cell carcinoma
Palopegteriparatide	Hypoparathyroidism	Danicopan	Extravascular hemolysis with paroxysmal nocturnal hemoglobinuria	Crovalimab-akkz	Paroxysmal nocturnal hemoglobinuria
		*Eladocagene exuparvovec-tneq*	L-Amino acid decarboxylase (AADC) deficiency	Deuruxolitinib	Alopecia areata
		Elafibranor	Primary biliary cholangitis	Donanemab-azbt	Alzheimer’s disease
		Givinostat	Duchenne muscular dystrophy	Ensartinib	Non-small cell lung cancer
		*Hematopoietic progenitor cell*	Hematopoietic system disorders	Ensifentrine	Chronic obstructive pulmonary disease
		Imetelstat	Low- to intermediate-1 risk myelodysplastic syndromes	*Fidanacogene elaparvovec-dzkt*	Moderate to severe hemophilia B
		Levacetylleucine	Niemann-Pick disease type C	Flurpiridaz F-18	Radioactive diagnostic drug for myocardial ischemia and infarction
		*Lifileucel*	Unresectable or metastatic melanoma	Inavolisib	Locally advanced or metastatic breast cancer
		Marstacimab-hncq	Hemophilia A or B	Iomeprol	Diagnostic radiographic contrast agent
		Nemolizumab	Prurigo nodularis	Landiolol	Supraventricular Tachycardia
		Nogapendekin alfa inbakicept-pmln	Bladder cancer	Lazertinib	Non-small cell lung cancer
		Pegulicianine	Optical imaging agent for the detection of cancerous tissue	Lebrikizumab-lbkz	Atopic dermatitis
		*Remestemcel-L-rknd*	Steroid-refractory acute graft-versus-host disease	LetibotulinumtoxinA-wlbg	Appearance of moderate-to-severe glabellar lines
		Resmetirom	Noncirrhotic non-alcoholic steatohepatitis	*Obecabtagene autoleucel*	Relapsed or refractory B-cell precursor acute lymphoblastic leukemia
		Revumenib	Relapsed or refractory acute leukemia	Seladelpar	Primary biliary cholangitis
		Sotatercept	Pulmonary arterial hypertension	Sofpironium	Primary axillary hyperhidrosis
		Tarlatamab-dlle	Extensive stage small cell lung cancer	Sulopenem etzadroxil, probenecid	Uncomplicated urinary tract infections
		Xanomeline and trospium chloride	Schizophrenia	Tislelizumab-jsgr	Unresectable or metastatic esophageal squamous cell carcinoma
		Zanidatamab-hrii	HER2-positive biliary tract cancer	Tovorafenib	Relapsed or refractory pediatric low-grade glioma
		Zenocutuzumab-zbco	Non-small cell lung cancer and pancreatic adenocarcinoma	Vadadustat	Anemia due to chronic kidney disease
		Zolbetuximab-clzb	Gastric or gastroesophageal junction adenocarcinoma	Vanzacaftor, tezacaftor, and deutivacaftor	Cystic fibrosis
				Vorasidenib	Grade 2 astrocytoma or oligodendroglioma

**Table 2:** Newly approved drugs grouped by drug type. Percentages are those of small molecules, antibody, peptides and proteins, nuclear acid based, cellular and gene therapies, with all drugs approved by the FDA in 2024 taken as 100%

Small molecule (*n*=31, 52.5%)	Antibody (*n*=13, 22.0%)	Peptide and protein (*n*=4, 6.8%)	Nucleic acid based (*n*=2, 3.4%)	Cellular and gene products (*n*=9, 15.3%)
Acoramidis	Axatilimab-csfr	LetibotulinumtoxinA-wlbg	Imetelstat	Afamitresgene autoleucel
Aprocitentan	Concizumab-mtci	Nogapendekin alfa inbakicept-pmln	Olezarsen	Atidarsagene autotemcel
Arimoclomol	Cosibelimab-ipdl	Palopegteriparatide		Eladocagene exuparvovec-tneq
Berdazimer	Crovalimab-akkz	Sotatercept-csrk		Fidanacogene elaparvovec-dzkt
Cefepime, enmetazobactam	Donanemab-azbt			Hematopoietic progenitor cell
Ceftobiprole	Lebrikizumab-lbkz			Lifileucel
Crinecerfont	Marstacimab-hncq			Obecabtagene autoleucel
Danicopan	Nemolizumab-ilto			Olezarsen
Deuruxolitinib	Tarlatamab-dlle			Remestemcel-L-rknd
Elafibranor	Tislelizumab-jsgr			
Ensartinib	Zanidatamab-hrii			
Ensifentrine	Zenocutuzumab-zbco			
Flurpiridaz F-18	Zolbetuximab-clzb			
Givinostat				
Inavolisib				
Iomeprol				
Landiolol				
Lazertinib				
Levacetylleucine				
Mavorixafor				
Pegulicianine				
Resmetirom				
Revumenib				
Seladelpar				
Sofpironium				
Sulopenem				
Tovorafenib				
Vadadustat				
Vanzacaftor, tezacaftor, and deutivacaftor				
Vorasidenib				
Xanomeline and trospium chloride

**Table 3:** Orphan drugs approved by the FDA in 2024. Percentage is that of orphan drugs within all drugs approved by the FDA in 2024 taken as 100%. Cellular and gene products are depicted in italics. https://www.accessdata.fda.gov/scripts/opdlisting/oopd/listResult.cfm

Drug (*n*=32, 54.3%)	Major indications
Acoramidis	Transthyretin amyloidosis
*Afamitresgene autoleucel*	Unresectable or metastatic synovial sarcoma
Arimoclomol	Niemann-Pick disease type C
*Atidarsagene autotemcel*	Metachromatic leukodystrophy
Axatilimab-csfr	Chronic graft-versus-host disease
Concizumab-mtci	Hemophilia A and B
Crinecerfont	Congenital adrenal hyperplasia
Crovalimab-akkz	Paroxysmal nocturnal hemoglobinuria
Danicopan	Paroxysmal nocturnal hemoglobinuria
Elafibranor	Primary biliary cholangitis
*Fidanacogene elaparvovec-dzkt*	Hemophilia B
Givinostat	Duchenne muscular dystrophy
Imetelstat	Myelodysplastic syndromes
Levacetylleucine	Niemann-Pick disease type C
*Lifileucel*	Malignant melanoma stages IIb to IV
Marstacimab-hncq	Hemophilia A and B
Mavorixafor	WHIM syndrome
*Obecabtagene autoleucel*	Acute lymphoblastic leukemia
Olezarsen	Familial chylomicronemia syndrome
Palopegteriparatide	Hypoparathyroidism
*Remestemcel-L-rknd*	Steroid-refractory acute graft-versus-host disease
Revumenib	Relapsed or refractory acute leukemia
Seladelpar	Primary biliary cholangitis
Sotatercept-csrk	Pulmonary arterial hypertension
Tarlatamab-dlle	Small cell lung cancer
Tislelizumab-jsgr	Esophageal squamous cell carcinoma
Tovorafenib	Relapsed or refractory pediatric low-grade glioma
Vanzacaftor, tezacaftor, and deutivacaftor	Cystic fibrosis
Vorasidenib	Grade 2 astrocytoma or oligodendroglioma
Zanidatamab-hrii	Biliary tract cancer
Zenocutuzumab-zbco	Pancreatic adenocarcinoma
Zolbetuximab-clzb	Gastric or gastroesophageal junction adenocarcinoma

It is explicitly not our intention to compare novel treatments with their specific advantages and disadvantages with existing ones, because this is best done by experts in a specific therapeutic area. Similarly, we do not discuss drug pricing for novel treatments: Such discussion can only be meaningful based on input from experts within a specific therapeutic area who can judge on the added clinical value of a treatment. They fall into the responsibility of Health Technology Assessment bodies such as the National Institute for Health and Care Excellence in the UK.

## Methods

Our analyses follow the same protocol as those for newly approved drugs in 2020–2023 (Kayki-Mutlu and Michel 2021, Kayki-Mutlu et al. 2022, Kayki-Mutlu et al. 2023, Kayki-Mutlu et al. 2024). We do not consider generics, or generic versions of biopharmaceuticals (“biosimilars”), and already approved drugs that received marketing authorizations for one or more additional indications and/or in a novel formulation. Newly approved drug combinations were only considered if at least one of the combination partners (mostly therapeutic antibodies) is a novel chemical or biopharmaceutical entity. We would like to emphasize that other regulatory agencies may have approved the same compounds earlier than the FDA (among this year’s approvals, e.g., eladocagene in the EU, nemolizumab and vadadustat in Japan, or lazertinib in Korea), may do so at later points in time, may choose not to approve some of these compounds, or may choose to approve compounds not approved by the FDA. Our focus on drug approvals by the FDA does not imply any opinion on the scientific quality of approvals by the FDA as compared to the regulatory authorities in other jurisdictions, but rather uses the FDA as a point of reference, due to its status as one of the most influential drug regulatory authorities. All indications refer to adults unless stated otherwise.

## Oncology

Esophageal squamous cell carcinoma is the most prevalent form of esophageal cancer worldwide and has a poor prognosis (Morgan et al. 2022). **Tislelizumab-jsgr**, a humanized monoclonal antibody that blocks programmed death receptor-1 (PD-1), has been approved for unresectable or metastatic esophageal cancer in adults. The interaction of PD-L1 and PD-L2 ligands with PD-1 receptors—inhibitory immune-modulatory receptors—decreases the anti-cancer immune response (Chen et al. 2019). Tislelizumab enhances anti-cancer immune responses by preventing ligand binding to the PD-1 receptor (Lee and Keam 2020). As indicated in the prescribing information, the most frequently observed adverse effects (AEs) associated with tislelizumab are hyperglycemia, a reduction in hemoglobin/occurrence of anemia, a decline in lymphocytes, hyponatremia, a decrease in albumin, elevated liver enzyme levels, fatigue, muscle pain, weight loss, and cough.

Bladder cancer is the 10th most prevalent cancer (Sung et al. 2021) and it is classified as non-muscle invasive and invasive bladder carcinoma. The non-muscle invasive bladder cancer has a poor prognosis and is more common (Chang et al. 2016). The gold standard treatment of this type is Bacillus Calmette-Guérin (BCG) therapy (Waheed et al. 2024). **Nogapendekin alfa inbakicept-pmln** received approval for utilization in conjunction with BCG for the management of BCG-unresponsive non-muscle invasive bladder cancer with carcinoma in situ, in the presence or absence of papillary tumors (Keam 2024f). It was granted the fast track and breakthrough designation (for definitions of these terms as used by the FDA, see 3^rd^ paragraph of the “General trends and conclusions” section). Nogapendekin alfa inbakicept is a first-in-class interleukin-15 receptor super-agonist fusion protein that produces immunotherapeutic effects by binding to its receptor, thereby increasing and activating natural killer and CD8^+^ T cells (Chen et al. 2022). The most commonly observed AEs include elevated creatinine levels, urinary disorders, hyperkalemia, pain, chills, and fever (Keam 2024f).

Gliomas are a substantial proportion of malignant primary brain tumors observed in adults and they are commonly associated with mutations in the isocitrate dehydrogenase 1 (IDH1) and IDH2 enzymes (Ruda et al. 2024). These enzyme mutations lead to increased formation of the oncometabolite D-2-hydroxyglutarate in the Krebs cycle (Ruda et al. 2024). **Vorasidenib** is a dual inhibitor of mutant IDH1/2 enzymes and is an orally available small-molecule drug that penetrates the blood-brain barrier (Mellinghoff et al. 2021). Vorasidenib has been approved as a systemic therapy for grade 2 astrocytoma or oligodendroglioma in patients (aged ≥12 years) with a susceptible IDH1/2 mutation, who have undergone surgical intervention (Lamb 2024). In addition, vorasidenib has been granted priority review, breakthrough therapy, fast track, and orphan drug designations (Lamb 2024). According to the prescribing information, the most common AEs were tiredness, seizures, SARS-CoV-2 infections, pain, and gastrointestinal disorders. Pediatric low-grade gliomas are the most common central nervous system tumors in children (Fangusaro et al. 2024). Many patients demonstrate variations in the mitogen-activated protein kinase signaling pathway, including the presence of a BRAF V600E mutation or BRAF fusion (Dhillon 2024d). **Tovorafenib** is a small molecule, brain-penetrant, selective type II RAF kinase inhibitor that targets mutant BRAF V600E, wild-type BRAF, wild-type CRAF kinases, and BRAF fusions (Dhillon 2024d). The paradoxical induction of mitogen-activated protein kinase signaling by type I BRAF inhibitors was identified, whereas this did not occur with the type II inhibitor tovorafenib (Sun et al. 2017, Khoury et al. 2024). Tovorafenib has been granted approval for the administration to patients with a minimum age of 6 months who have experienced relapsed or refractory pediatric low-grade glioma with a confirmed presence of a BRAF fusion or rearrangement, or with a BRAF V600 mutation (Dhillon 2024d). This medication received accelerated approval, priority review, breakthrough therapy, and rare pediatric disease designations (Dhillon 2024d, Yaman and Bouffet 2024). Tovorafenib is an acceptable safety profile (van Tilburg et al. 2024). The most common AEs were alterations in hair color, increased creatine phosphokinase levels, anemia, tiredness, and rash (Dhillon 2024d).

Lung cancer is one of the most prevalent and life-threatening cancers, with the majority of cases identified as non-small cell lung cancer as compared to small cell lung cancer (Goyal and Sangwan 2024). However, small cell lung cancer is an aggressive and metastatic neuroendocrine carcinoma with a high propensity for extensive disease at diagnosis, which is associated with a poor prognosis (Saida et al. 2023). **Tarlatamab-dlle** has been approved for the therapeutic management of extensive-stage small cell lung cancer in adults who have exhibited clinical progression of the disease, or following the administration of platinum-based chemotherapy (Dhillon 2024c). Tarlatamab binds to delta-like ligand 3 (DLL3) overexpressed in the majority of small cell lung cancer cells and CD3 expressed on the T cells, resulting in the lysis of DLL3-expressing cells, activation of T cells, and the subsequent release of inflammatory cytokines (Dhillon 2024c, Goyal and Sangwan 2024). Tarlatamab represents a first-in-class therapeutic agent that employs a bispecific T cell engager with an extended half-life (Dhillon 2024c). It has received accelerated approval, priority review, breakthrough therapy, and orphan drug designation. This medication has a black box warning about cytokine release syndrome and neurologic toxicity. The most commonly reported AEs were cytokine release syndrome, tiredness, fever, altered sense of taste, loss of appetite, pain, constipation, anemia, and nausea, as described in the prescribing information.

The FDA’s approval of a second medication for lung cancer in 2024 has been granted to **lazertinib**, which had previously been approved in the Republic of Korea in 2021 (Dhillon 2021). This drug was granted priority review designation. Lazertinib has been approved for the initial therapy of locally advanced or metastatic non-small cell lung cancer exhibiting epidermal growth factor receptor (EGFR) exon 19 deletions or exon 21 L858R substitution mutations in adults, in combination with amivantamab. Amivantamab, a bispecific monoclonal antibody, was approved by the FDA in 2021 for non-small cell lung cancer with an EGFR exon 20 insertion mutation (Kayki-Mutlu et al. 2022). EGFR-tyrosine kinase inhibitors are first-line medications for advanced or metastatic non-small cell lung cancer harboring an EGFR mutation (Chul Cho et al. 2024). Lazertinib is a third-generation potent tyrosine kinase inhibitor that is permeable to the brain and irreversibly and selectively inhibits the EGFR (Dhillon 2021). The most commonly reported AEs included pain, swelling, venous thromboembolism, tiredness, infection with the SARS-CoV-2 virus, bleeding, loss of appetite, and gastrointestinal, ocular, and dermatological disorders, based on the prescribing information. The third medication that has been authorized for lung cancer is **zenocutuzumab-zbco**, which is also approved for pancreatic adenocarcinoma. Zenocutuzumab-zbco is a first-in-class, bispecific antibody designed to recognize and bind to both human epidermal growth factor receptor (HER) 2 and HER3 proteins, thereby preventing HER2:HER3 dimerization and blocking neuregulin 1 binding to HER3 (Schram et al. 2022, Kim et al. 2024). Neuregulin 1 fusions are a rare type of oncogenic driver, but their potential as a target for precision therapy in solid cancers is noteworthy (Liu 2021). Zenocutuzumab has received approval for the management of advanced, unresectable or metastatic non-small cell lung cancer and metastatic pancreatic adenocarcinoma that harbor a neuregulin 1 gene fusion with disease progression, or following previously administered systemic therapy in adults as first-in-class. Zenocutuzumab was granted accelerated approval, priority review, fast track, breakthrough, and orphan drug designations. A black box warning has been included due to the potential risk of embryo-fetal toxicity. The most common AEs were gastrointestinal disorders, pain, tiredness, infusion-related reactions, breathlessness, rash, and fluid retention, according to prescription information. The fourth approved medication for lung cancer is **ensartinib**, which is a potent, second-generation anaplastic lymphoma kinase (ALK) inhibitor (Li et al. 2019, Spitaleri et al. 2019). A subset of patients diagnosed with non-small cell lung cancer exhibit a translocation in the ALK gene (Chia et al. 2014), and ensartinib is a small tyrosine kinase inhibitor targeting this proto-oncogene. Ensartinib has been approved for ALK-positive non-small cell lung cancer in adults who have not undergone treatment with an ALK inhibitor. Treatment with ersartinib was well-tolerated, and the most common AEs were gastrointestinal disorders, pruritus, rash, cough, and tiredness (Li et al. 2019).

Myelodysplastic syndromes constitute a heterogeneous group of malignancies (Garcia-Manero 2023). Many patients are diagnosed with low or intermediate risk, and the prevalence of anemia among these patients is high (Platzbecker et al. 2024). In patients with myelodysplastic syndrome, there is an increase in telomerase activity and a reduction in telomere length in bone marrow cells, which is associated with a poor prognosis (Park et al. 2017). Telomerase is the target of cancer therapy, since most cancer and neoplastic progenitor cells exhibit elevated levels of telomerase expression, which facilitates cellular immortality and the maintenance of telomeres (Lennox et al. 2024). Thus, telomerase activity is emerging as a potential therapeutic target (Platzbecker et al. 2024). **Imetelstat** is a first-in-class, direct, and competitive oligonucleotide inhibitor of telomerase enzymatic activity (Lennox et al. 2024). Imetelstat has been approved for low- to intermediate-risk myelodysplastic syndromes with transfusion-dependent anemia (Keam 2024d). Imetelstat is recommended for use in patients who require four or more red blood cell units for 8 weeks and who are unresponsive or have demonstrated a loss of response to, or who are ineligible for, erythropoiesis-stimulating agents (Keam 2024d). It has been granted fast track and orphan drug designation. The most commonly observed AEs include laboratory abnormalities, tiredness, an extended partial thromboplastin time, and pain (Keam 2024d).

Hormone receptor (HR)-positive and human epidermal growth factor receptor 2 (HER2)-negative breast cancer is the most prevalent breast cancer subtype (Giaquinto et al. 2024). The presence of phosphatidylinositol-3-kinase complex (PIK3CA) mutations in patients with HR^+^ advanced breast cancers has a poor prognosis (Turner et al. 2024). **Inavolisib** is a potent and selective small molecule inhibitor of the p110α catalytic subunit of the PI3K complex and induces the cleavage of mutated p110α (Jhaveri et al. 2024, Turner et al. 2024). This kinase inhibitor has received approval for the therapeutic management of endocrine-resistant, PIK3CA-mutated, HR^+^/HER2^−^, locally advanced or metastatic breast cancer in adults. This therapy has received breakthrough therapy and priority review designations. Inavolisib is recommended for use in combination with palbociclib, a selective cyclin-dependent kinases 4 and 6 inhibitor, and fulvestrant, an estrogen receptor antagonist, in cases where recurrence occurs on or after completing adjuvant endocrine therapy. Inavolisib has a manageable safety profile, and the most common AEs were laboratory abnormalities, gastrointestinal disorders, tiredness, rash, and pain (Turner et al. 2024).

Stomach cancer is characterized by low survival, due to the limited rate of early diagnosis and the elevated risk of recurrence following treatment (Peixoto and Donadio 2024). **Zolbetuximab-clzb** was approved for the indication of gastric or gastroesophageal junction adenocarcinoma. Zolbetuximab is a first-in-class, recombinant, chimeric, claudin 18.2 (CLDN 18.2)-directed cytolytic monoclonal antibody (Keam 2024g). The tight junction protein family member CLDN18.2 was the target of zolbetuximab treatment due to its overexpression on the tumor cells (Peixoto and Donadio 2024). Zolbetuximab is indicated for locally advanced unresectable or metastatic HER2-negative and CLDN 18.2-positive gastric or gastroesophageal junction adenocarcinoma in combination with fluoropyrimidine- and platinum-containing chemotherapy in adults as the first-line therapy. Zolbetuximab has been granted priority review, fast track, and orphan drug designation (Keam 2024g). The most common AEs associated with these combinations were tiredness, loss of appetite and weight, peripheral neuropathy, gastrointestinal disorders, hypersensitivity, and fever, as documented in the prescribing information.

Acute leukemia is characterized by a disruption of differentiation and uncontrolled cell proliferation in hematopoietic cells caused by genetic changes (Salman and Stein 2024). Acute leukemias that harbor lysine methyltransferase 2A (KMT2A) gene rearrangements are linked to poor prognosis and chemotherapy resistance, and menin is a key oncogenic cofactor in this type of leukemia (Issa et al. 2023). **Revumenib** is a first-in-class, potent, and selective menin inhibitor and impedes the interaction between KMT2A and menin (Issa et al. 2023). Revumenib has been approved in adult and pediatric patients aged ≥ 1 year with relapsed or refractory acute leukemia with KMT2A gene translocations. This medication has been granted priority review, fast track, breakthrough, and orphan drug designations. A black box warning has been issued for revumenib about the potential for differentiation syndrome to progress to a fatal outcome. The dose-limiting toxicity of revumenib is a prolongation of the QT interval on electrocardiography (Issa et al. 2023). In addition, the most common AEs listed in the prescribing information are laboratory abnormalities, infection, gastrointestinal disorders, loss of appetite, edema, and tiredness.

The development of chimeric antigen receptor (CAR)-T cell therapies has remarkably impacted treating lymphoid malignancies (Kopmar and Cassaday 2024). CAR-T cell therapies, including tisagenlecleucel (tisa-cel) for children and young adults and brexucabtagene autoleucel (brexu-cel) for adults, were previously approved by the FDA for relapsed/refractory B-cell acute lymphoblastic leukemia (Roddie et al. 2024). ***Obecabtagene autoleucel*** (obe-cel) is an autologous anti-CD19 genetically modified CAR-T cell immunotherapy. In comparison to other CAR-T cell therapies, obe-cel is designed to have a faster binding off-rate and has been suggested to produce less immunological toxicity and provide more durable CAR-T persistence (Roddie et al. 2021). This cell therapy has been approved for relapsed/refractory B-cell precursor acute lymphoblastic leukemia in adults. The prescription information contains a black box warning regarding cytokine release syndrome, neurological toxicities, and secondary hematological malignancies. In addition, the most common AEs were infections, pain, fever, gastrointestinal disorders, tiredness, febrile neutropenia, hypotension, encephalopathy, and hemorrhage.

Biliary tract cancers represent a small percentage of adult cancers, with the majority being diagnosed as incurable, locally advanced, or metastatic disease (Tella et al. 2020, Giaquinto et al. 2024). The amplification or overexpression of HER2 has been detected in a subset of patients with biliary tract cancer, indicating HER2 as a probable therapeutic target for this malignancy (Galdy et al. 2017). **Zanidatamab-hrii** is a first-in-class, humanized, bispecific, biparatopic monoclonal antibody and targets HER2 via two distinct antigen-binding sites, resulting in HER2 internalization and downregulation (Weisser et al. 2023). In addition, zanidatamab exerts an inhibitory effect on tumor cell proliferation and induces antibody-dependent cellular cytotoxicity, antibody-dependent cellular phagocytosis, and complement-dependent cytotoxicity (Proctor et al. 2022). This medication has been approved for use in cases of HER2-positive (IHC 3+) biliary tract cancer that has been treated previously and is unresectable or metastatic. Zanidatamab has been granted accelerated approval, fast track, priority review, breakthrough therapy, and orphan drug designations. The prescription information of zanidatamab has a black box warning about embryo-fetal toxicity. The most common AEs were diarrhea and infusion-related reactions (Harding et al. 2023).

Cutaneous squamous cell carcinoma represents the second most prevalent form of skin cancer, and the recurrence of the disease is a critical consideration in cases of locally advanced and metastatic disease (Clingan et al. 2023). This malignancy is marked by substantial expression of the PD-1/PD-L1 axis in both tumor tissues and tumor-infiltrating immune cells (Rubatto et al. 2022). The interaction between PD-L1, which is expressed in tumor cells, and PD-1, which is found in T cells, results in the suppression of the antitumor immune response (Wang et al. 2016). As a result, immunotherapeutic approaches that prevent the binding of PD-L1 to PD-1 have been developed. **Cosibelimab-ipdl** is a PD-L1 blocking monoclonal antibody with a high affinity (Clingan et al. 2023). The medication has been granted approval for the management of metastatic or locally advanced cutaneous squamous cell carcinoma in adults who are not eligible for curative operations or radiotherapy. In a phase 1 study conducted on patients diagnosed with metastatic cutaneous squamous cell carcinoma, cosibelimab demonstrated a tolerable safety profile (Clingan et al. 2023). The most common AEs were tiredness, pain, rash, gastrointestinal disorders, decreased thyroid hormone levels, itching, fluid retention, and infections, according to the prescribing information.

Adoptive cell therapy using tumor-infiltrating lymphocytes (TILs) is a new therapeutic option for advanced melanoma. TILs can recognize specific tumor markers in the immune system and kill tumor cells, but this response is not obtained if the tumor microenvironment suppresses the immune response (Keam 2024e). In adoptive cell therapy with TILs, the TILs are isolated from the microenvironment of the patient’s tumor, expanded ex vivo, and re-infused into the patient (Zhao et al. 2022). ***Lifileucel*** is a first-in-class, tumor-derived, adoptive cell therapy with autologous TIL (Parums 2024). This cell-based immunotherapy is received approval for the indication of unresectable or metastatic melanoma in previously treated adults (Keam 2024e). Lifileucel was granted accelerated approval, priority review, fast track, orphan drug, and regenerative medicine advanced therapy designations (Keam 2024e). The prescribing information of lifileucel has been a black box warning about mortality, cytopenia, infection, cardiopulmonary, and renal disorders. The most frequent AEs were chills, gastrointestinal and cardiovascular problems, fever, tiredness, febrile neutropenia, edema, rash, reduced appetite, hair loss, infection, and pain (Keam 2024e).

Soft tissue sarcomas are among the rare cancers, and elevated levels of cancer testicular antigens are detected (Keam 2024a). Melanoma-associated antigen 4 (MAGE-A4), an intracellular cancer testis antigen expressed in synovial sarcoma, is emerging as a target for therapeutic intervention (Wang et al. 2024). ***Afamitresgene autoleucel*** is a genetically engineered human leukocyte antigen (HLA)-restricted autologous MAGE-A4-directed T cell immunotherapy (D’Angelo et al. 2024). This first-in-class cell therapy is developed by enriching peripheral blood mononuclear cells obtained from the patient for T cells and transducing them with a lentiviral vector containing the MAGEA4 T cell receptor transgene (Keam 2024a). In exploratory analyses from a phase 1 study, afamitresgene autoleucel was shown to penetrate tumors, exhibit an interferon-γ-driven mechanism of action, and induce adaptive immune responses (Hong et al. 2023). It has been approved to manage unresectable or metastatic synovial sarcoma in previously treated adults. Patients who meet the eligibility criteria for receiving this medication include those who are HLA-A*02:01P, -A*02:02P, -A*02:03P, or -A*02:06P positive, and whose tumor expresses the MAGE-A4 antigen. Afamitresgene autoleucel was granted accelerated approval, regenerative medicine advanced therapy, priority review, and orphan drug designation (Keam 2024a). The prescribing information contains a black box warning regarding the common risk of cytokine release syndrome. Additionally, the most common AEs included gastrointestinal and cardiovascular disorders, laboratory abnormalities, tiredness, infections, fever, pain, reduced appetite, and fluid retention (Keam 2024a).

## Hematology/immunology

Renal anemia, a comorbid condition with advanced chronic kidney disease, has been linked to the progression of both chronic heart failure and chronic kidney disease (Silverberg et al. 2006). The treatment of renal anemia currently includes the hypoxia-inducible factor prolyl-hydroxylase inhibitors and erythropoiesis-stimulating agents (Toka et al. 2024). Hypoxia-induced factor (HIF) prolyl-hydroxylase inhibitors (roxadustat, daprodustat, vadadustat, enarodustat, and molidustat) enhance erythropoietin production and have been approved in Japan for renal anemia (Imai and Imai 2024). Among these drugs, daprodustat was granted approval by the FDA as a first-in-class medication last year (Kayki-Mutlu et al. 2024), while **vadadustat** received approval this year for the management of anemia resulting from chronic renal failure in adults who have previously undergone dialysis for a minimum of 3 months. Vadadustat is an oral, reversible inhibitor of HIF-specific prolyl-hydroxylase inhibitors and stabilizes the HIF (Chertow et al. 2021). Vadadustat has a black box warning about thrombotic vascular events. The most common AEs were elevations in blood pressure and gastrointestinal disorders (Markham 2020).

Paroxysmal nocturnal hemoglobinuria (PNH) is a rare, acquired dyscrasia that affects the complement system, resulting in an augmented risk of thrombosis, hemolytic anemia, bone marrow failure, and organ damage (Parker et al. 2005, Kulasekararaj and Lazana 2023). The conventional therapeutic strategy for this condition is complement component 5 (C5) inhibition with eculizumab or ravulizumab; however, this approach is associated with several undesirable effects, such as hemolysis (Kulasekararaj and Lazana 2023). In 2024, the FDA approved two medications for the management of PNH. The first was **danicopan**, which is a first-in-class, oral, reversible complement factor D inhibitor and approved for extravascular hemolysis with paroxysmal nocturnal hemoglobinuria in combination with eculizumab or ravulizumab in adults (Fahim et al. 2024b). Danicopan has been granted orphan drug and breakthrough therapy designation (Kang 2024a). Danicopan is marked with a black box warning, indicating a high risk of infection with *Neisseria meningitidis*, *Streptococcus pneumoniae*, and *Haemophilus influenzae* type B*.* The most frequent AEs included headache, pyrexia, abdominal discomfort, and hepatic impairment (Kang 2024a). The second medication is **crovalimab-akkz**, a complement C5 inhibitor, has been approved for the management of PNH in patients over the age of 13 and weighing ≥40 kg. Crovalimab is a humanized, recycling monoclonal antibody that has been designed utilizing the Sequential Monoclonal Antibody Recycling Technology (SMART-Ig) for the purpose of prolonging the half-life, enhancing the bioavailability and solubility (Liu et al. 2023, Scheinberg et al. 2024). Therefore, crovalimab induces persistent complement inhibition through repeated binding to the relevant antigen (Dhillon 2024a). The most common AEs were infusion-related reactions, infections, and type III hypersensitivity reactions, according to prescribing information. Similar to danicopan, crovalimab also has a black box warning about *Neisseria meningitidis* infection.

Graft-versus-host disease is a rare orphan disease and a common complication following allogeneic hematopoietic cell transplantation (Tremblay et al. 2021). Colony-stimulating factor 1 receptor-dependent monocytes and macrophages induce inflammation, tissue injury, and fibrosis in graft-versus-host disease (Kitko et al. 2023, Keam 2024b). Hence, the colony-stimulating factor 1 receptor has been a therapeutic target to inhibit signaling pathways involved in inflammation and fibrosis in chronic graft-versus-host disease. **Axatilimab-csfr** is a humanized monoclonal antibody directed against the colony-stimulating factor-1 receptor and diminishes the levels of proinflammatory and profibrotic monocytes and macrophages in the circulation (Keam 2024b). Axatilimab has been granted fast track, priority review, and orphan drug designations and is approved to treat chronic graft-versus-host disease following a minimum of two systemic therapy failures in adult and pediatric patients who weighed over 40 kg (Keam 2024b). The most common AEs were laboratory abnormalities, infection, pain, tiredness, fever, and gastrointestinal and respiratory disorders (Keam 2024b). In addition, ***remestemcel*-L-rknd**, a first-in-class bone marrow-derived mesenchymal stromal cell product (Chen et al. 2014), has been approved for the management of acute graft-versus-host disease in pediatric patients (≥ 2 months) who are steroid-refractory. Acute graft-versus-host disease in patients who are unresponsive to steroid therapy exhibits a high mortality risk (Tremblay et al. 2021). The efficacy of remestemcel-L is attributable to its immunomodulatory properties (Chen et al. 2014). Remestemcel-L has been granted priority review, fast track, and orphan drug designations. In phase 3 studies, remestemcel-L had an acceptable safety and tolerability profile (Kebriaei et al. 2020, Kurtzberg et al. 2020). The most common AEs were infections, fever, hemorrhage, edema, pain, and hypertension, according to prescribing information.

Hemophilia A and B are rare, congenital coagulation diseases characterized by a deficiency of the FVIII and FIX, respectively (Mahlangu et al. 2023a). In a subset of hemophilia patients exhibiting severe symptoms, prophylactic treatment with these factors fails to prevent bleeding and joint disease (Mancuso et al. 2024). In addition, developing neutralizing antibodies in some patients in response to replacement therapy with clotting factors necessitates for nonfactor therapies (Arruda et al. 2017, Mahlangu et al. 2023b). In 2024, two anti-tissue factor pathway inhibitors humanized monoclonal antibodies, **marstacimab-hncq and concizumab-mtci**, were approved by the FDA, respectively, as nonfactor therapy for patients with hemophilia A and B. Tissue factor pathway inhibitor antagonizes initial coagulation phases by inhibiting FVIIa and FXa (Mahlangu et al. 2023a). Marstacimab and concizumab targeting the tissue factor pathway inhibitor result in the suppression of its activity and the enhancement of coagulation (Mancuso et al. 2024). These monoclonal antibodies have been approved for the routine prophylaxis of bleeding incidents in patients aged 12 years and over with hemophilia A and B. Marstacimab has been granted orphan drug designation, and concizumab received priority review and orphan drug designations for hemophilia A and B. According to the prescribing information, the most frequent AEs were injection-site reactions, pain, and urticaria with marstacimab, and injection-site reactions and urticaria with concizumab. Furthermore, the FDA approved a gene therapy, ***fidanacogene elaparvovec*-dzkt**, for the prophylactic treatment of hemophilia B in 2024. Fidanacogene elaparvovec is a nonreplicating, recombinant adeno-associated virus vector expressing the high-activity human FIX (Pittman et al. 2024). It was approved to treat moderate to severe hemophilia B in eligible adults. This gene therapy is indicated for patients receiving factor IX prophylaxis or who have a history of life-threatening hemorrhage or recurrent, severe spontaneous hemorrhagic incidents and who remain negative for neutralizing antibodies to the adeno-associated virus serotype Rh74var capsid (Dhillon 2024b). Fidanacogene elaparvovec has been granted breakthrough therapy, orphan drug, and regenerative medicine status (Dhillon 2024b). This gene therapy is not designated for use in female patients (Dhillon 2024b). The most frequent AEs were laboratory abnormalities, pain, and infection (Dhillon 2024b).

In 2024, a pivotal development occurred with the approval of ***hematopoietic progenitor cell* (HPC)** as the first commercial vital cord blood stem cell therapy product (US Food and Drug Administration 2024). The efficacy of the product has been demonstrated by the single-arm prospective COBLT study, FDA data, and retrospective analyses of data from an observational database (US Food and Drug Administration 2024). This allogeneic human umbilical cord blood-derived hematopoietic progenitor cell therapy has been approved for administration in unrelated donor hematopoietic progenitor cell transplantation for patients diagnosed with hematological disorders (US Food and Drug Administration 2024). The prescribing information for the product includes a black box warning regarding fatal infusion reactions, graft versus host disease, engraft syndrome, and graft failure. As stated in the prescribing information, the most common AEs are hypertension, abdominal discomfort, bradycardia, and pyrexia.

## Neurology

Two anti-amyloid monoclonal antibodies, aducanumab and lecanemab, were approved by the FDA for Alzheimer’s disease in 2021 and 2023, respectively (Kayki-Mutlu et al. 2022, Kayki-Mutlu et al. 2024). In 2024, **donanemab-azbt** was approved as a third amyloid beta-directed humanized monoclonal antibody. Nevertheless, despite the evidence that these anti-amyloid antibodies markedly reduce amyloid in the brain, there is a controversy regarding the reliability of the evidence that these medications decelerate cognitive decline (Kurkinen 2023, Hoilund-Carlsen et al. 2024). Donanemab has been approved to treat early symptomatic Alzheimer’s disease and the eligibility criteria for treatment with this medication are constrained to patients with mild cognitive disorder or in the mild dementia phase (Kang 2024b). This medication has been granted fast track, breakthrough therapy, and priority review designations (Kang 2024b). Donanemab has a black box warning about life-threatening amyloid-related imaging abnormalities. The most common AEs were amyloid-related imaging abnormalities with edema or hemosiderosis and microhemorrhage, pain, and infusion reactions (Kang 2024b).

Schizophrenia is a multifaceted psychotic disorder characterized by symptoms including hallucinations, delusions, and cognitive impairments (Jauhar et al. 2022). Its pathophysiology is attributed to neurochemical disruptions within the dopaminergic and glutamatergic systems (Jauhar et al. 2022). The dopamine receptor blockers represent the main class of drugs used to treat schizophrenia; however, treatment resistance remains a substantial challenge (Vasiliu et al. 2024). A new antipsychotic combination comprising **xanomeline**, a muscarinic agonist, and **trospium**, a peripheral muscarinic antagonist, has been approved to treat schizophrenia in adults (Hasan and Abid 2024). This first-in-class medication provides symptomatic relief for patients with schizophrenia, in addition to mitigating the adverse effects of dopamine blockers, including weight increase or motor dysfunction (Hasan and Abid 2024). Xanomeline-trospium combination was found to be well tolerated and the most common AEs were gastrointestinal disorders, dizziness, pain, and hypertension (Kaul et al. 2024a, Kaul et al. 2024b).

Metachromatic leukodystrophy is a rare, hereditary, progressive neurodegenerative disease due to a lysosomal storage defect resulting from recessive mutations in the gene encoding arylsulfatase-A (Fahim et al. 2024a). A decrease in the activity of this enzyme leads to increased levels of sulfatides within the nervous system (van Rappard et al. 2015). This fatal disease is marked by motor and cognitive dysfunction and is classified into three distinct types based on the age of symptoms onset: late infantile, juvenile (early/late), and adult (Eichler et al. 2022). **Atidarsagene autotemcel** or arsa-cel is a single-use, autologous hematopoietic CD34^+^ stem cell-based gene therapy targeting the arylsulfatase-A gene (Armstrong et al. 2023, Fahim et al. 2024a). These stem cells have been genetically modified by the lentiviral vector, resulting in the expression of a functional form of the ARSA enzyme (Armstrong et al. 2023). Atidarsagene autotemcel has been approved as first-in-indication to treat metachromatic leukodystrophy in pre-symptomatic late infantile, pre-symptomatic early juvenile, or early symptomatic early juvenile stages in children. According to the prescription information, the most common AEs were febrile neutropenia, oral inflammation, infections, fever, rash, and hepatomegaly.

## Cardiovascular and respiratory disorders

In 2024, the first endothelin receptor antagonist was approved for the treatment of arterial hypertension in combination with other antihypertensive drugs. **Aprocitentan**, a dual endothelin A (ETA) and B (ETB) receptor antagonist, is administered orally. Previously, the endothelin pathway had not been a therapeutic target in arterial hypertension, despite its known role in the pathogenesis of the condition. Endothelin, a potent vasoconstrictor, contributes to endothelial dysfunction, increases the release of aldosterone and catecholamines, and promotes vascular remodeling (Tamargo 2024). Aprocitentan therapy effectively reduces systolic and diastolic blood pressure in patients with resistant hypertension (Schlaich et al. 2022). The most common AEs reported were oedema and fluid retention.

Another antihypertensive drug, **sotatercept-csrk**, was approved to treat pulmonary arterial hypertension. It is the first activin signaling inhibitor approved to enhance exercise capacity, upgrade WHO functional class, and lower the risk of clinical worsening. Sotatercept is a recombinant fusion protein that binds to activins and growth differentiation factors, helping to rebalance growth-promoting and inhibiting pathways (Humbert et al. 2021). Clinical trials have shown that sotatercept reduces pulmonary vascular resistance and enhances exercise capacity in patients with pulmonary hypertension (Humbert et al. 2021). It is administered subcutaneously. Common AEs include headache, epistaxis, rash, dizziness, telangiectasia, diarrhea, and erythema. There is also an increased risk of severe thrombocytopenia and erythrocytosis. The therapy has received breakthrough therapy, priority review, and orphan drug designations.

**Acoramidis** is a transthyretin stabilizer approved for treating cardiomyopathy caused by wild-type or variant transthyretin-mediated amyloidosis (ATTR-CM) in adults, with the goal of reducing cardiovascular mortality and hospitalization. It is the second therapy approved for this condition. ATTR-CM results from the accumulation of misfolded monomeric transthyretin in the heart. Acoramidis works by inhibiting dissociation of tetrameric TTR, thereby stabilizing it (Gillmore et al. 2024). Acoramidis improves key outcomes, including all-cause mortality, cardiovascular-related hospitalization, NT-proBNP, and 6-min walking distance (Gillmore et al. 2024). Common mild AEs include abdominal pain and diarrhea. Serious AE, such as cardiac disorders (including cardiac failure and atrial fibrillation), infections, and gastrointestinal issues, have also been reported. Acoramidis has been granted orphan drug designation.

An ultra short-acting selective β_1_-adrenoceptor antagonist, **landiolol**, has been approved for the short-term management of ventricular rate in patients experiencing supraventricular tachycardia. It is administered via intravenous infusion. Landiolol has been shown to reduce heart rate, along with minimal, dose-dependent negative inotropic effects in patients experiencing SVT in various conditions, including sepsis and acute decompensated HF (Nasoufidou et al. 2024). In septic shock patients, landiolol therapy reduced heart rate without increasing vasopressor use (Rehberg et al. 2024). The most frequent AEs reported were hypotension.

The approval of a bioengineering product, known as the **acellular tissue-engineered vessel-tyod**, in 2024 is significant in the field of regenerative medicine. This approval offers a potential solution for managing severe and potentially debilitating vascular injuries that may otherwise result in severe functional impairment, limb loss, or even fatality (Moore et al. 2024). This product, which is a sterile, off-the-shelf vascular conduit, is obtained from human vascular cells and then modified to be cell-free; thus, it can be implanted without necessitating immunosuppression (Moore et al. 2024). The findings from two open-label, single-arm, non-randomized clinical studies conducted in both civilian and military settings have demonstrated that the administration of acellular tissue engineered vessels to patients results in benefits with regard to patency, amputation, and infection (Moore et al. 2024). Acellular tissue-engineered vessel-tyod has been approved for the surgical replacement of damaged arterial vessels in adult patients as a first-in-indication. This product has received priority review and regenerative medicine advanced therapy. It has a black box warning about graft failure. According to prescribing information, the most common AEs were thrombosis, pyrexia, pain, anastomotic failure, and infection.

**Ensifentrine**, a selective dual inhibitor of phosphodiesterase 3 and 4, has been approved as a treatment for chronic obstructive pulmonary disease, exerting bronchodilator and anti-inflammatory effects. Treatment with ensifentrine improved dyspnea parameters including the Transition Dyspnea Index, Evaluating Respiratory Symptoms, and the use of rescue medication (Mahler et al. 2024). It also improves lung function (Forced Expiratory Volume-FEV), symptoms, and quality of life (Anzueto et al. 2023). Ensifentrine is administered twice daily via inhalation, with AEs including back pain, hypertension, urinary tract infection, and diarrhea.

## Endocrine and hepatobiliary diseases

**Palopegteriparatide** was approved as the first and only treatment for hypoparathyroidism in adults. It is a prodrug comprising parathyroid hormone (PTH) conjugated to a methoxy polyethylene glycol carrier. Upon subcutaneous administration, PTH is cleaved from the conjugated to maintain systemic PTH exposure, regulating calcium and phosphate metabolism. Palopegteriparatide therapy maintains normocalcemia without the need of conventional treatments, including vitamin D and calcium (Khan et al. 2023). It also improves renal function in patients with hypoparathyroidism (Rejnmark et al. 2024). Its AEs include reactions at the injection site, vasodilatory symptoms, headache, diarrhea, oropharyngeal and back pain, and hypercalcemia. This therapy has received orphan drug designation and priority review.

The FDA approved **resmetirom** for the treatment of noncirrhotic non-alcoholic steatohepatitis with moderate to advanced liver scarring (fibrosis). It is the first drug approved for this condition that is administered orally and used along with diet and exercise. The therapy has received accelerated approval, fast track, breakthrough therapy, and priority review. Resmetirom is a liver-directed selective thyroid hormone receptor-β agonist, regulating hepatic lipid metabolism and inflammation (Suvarna et al. 2024). The therapy improves liver fibrosis (Harrison et al. 2023) and is well tolerated, with AEs including diarrhea, nausea, and pruritus.

In 2024, two drugs were approved for primary biliary cholangitis, a rare, chronic liver disease causing bile duct destruction, cholestasis, and liver fibrosis. **Elafibranor**, a first-in-class drug, is an oral dual peroxisome proliferator-activated receptor (PPAR) α and β/δ agonist that inhibits bile acid synthesis. It is prescribed alongside ursodeoxycholic acid (UDCA) for patients who do not respond adequately to UDCA or as a standalone treatment for those who cannot tolerate UDCA (Blair 2024). Elafibranor improves biochemical indicators, including alkaline phosphatase levels (Kowdley et al. 2024). Reported AE include abdominal pain, diarrhea, nausea, and vomiting. The second drug approved for this condition, **seladelpar**, is also a PPAR-δ agonist. It normalizes alkaline phosphatase levels and reduces pruritus (Hirschfield et al. 2024). Its most frequent AEs were reported as headache, abdominal pain, nausea, abdominal distension, and dizziness. Both therapies have received accelerated approval, a designation as breakthrough therapy, and priority review.

## Infectious diseases

A combination of a 4th-generation cephalosporin, **cefepime**, and an extended-spectrum β-lactamase inhibitor, **enmetazobactam**, has been approved for treating patients with complicated urinary tract infections caused by multi-drug-resistant Gram-negative bacteria (Keam 2024c). This therapy has received fast track and priority review designations. In clinical trials, cefepime/enmetazobactam met the criteria for noninferiority versus piperacillin/tazobactam and demonstrated superiority in the primary outcomes of clinical cure and microbiological response (Kaye et al. 2022). It is administered intravenously for 7 to 14 days. Common AEs include increased transaminases and bilirubin, headache, and phlebitis/infusion site reactions.

**Ceftobiprole medocaril sodium**, a cephalosporin, has been indicated for three different uses: *Staphylococcus aureus* bloodstream infections (including right-sided infective endocarditis); acute bacterial skin and skin structure infections (ABSSSIs); and community-acquired pneumonia (CAP) in adults and pediatric patients aged 3 months and older. It is a prodrug of ceftobiprole, a broad-spectrum agent against both Gram-positive and Gram-negative bacteria. It is administered intravenously. Clinical trials reported noninferiority to daptomycin in patients with right-sided endocarditis (Holland et al. 2023); noninferiority to vancomycin/aztreonam in patients with ABSSSIs (Overcash et al. 2021); and noninferiority to ceftriaxone ± linezolid in treating CAP (Nicholson et al. 2012). Frequently reported AEs are nausea, elevated liver enzymes, vomiting, diarrhea, rash, insomnia, phlebitis, high blood pressure, and dizziness. This drug has received fast track and priority review designations.

The combination of **sulopenem etzadroxil**, a penem antibiotic, and **probenecid**, a renal tubular transport blocker, has been approved to treat uncomplicated urinary tract infections. It is the first oral penem drug and it has demonstrated superiority to ciprofloxacin in patients with ciprofloxacin-nonsusceptible pathogens, while being noninferior in those with ciprofloxacin-susceptible pathogens (Dunne et al. 2023). The most frequent AEs reported are diarrhea, nausea, vulvovaginal mycotic infection, and headache. This therapy has received fast track and priority review designations.

**Berdazimer** is indicated for treating molluscum contagiosum, a viral skin infection that spreads easily. It is a first-in-class topical gel suitable for self-application in both adults and pediatric patients. Berdazimer releases nitric oxide, acting as a short-lived immune modulator and a broad-spectrum antimicrobial agent (Stasko et al. 2018). Clinical trials have demonstrated its efficacy and safety (Sugarman et al. 2024). The most frequent AEs include site reactions, pain, erythema, and pruritus.

## Genetic disorders

**Givinostat**, an oral drug, is indicated for treating Duchenne muscular dystrophy in individuals aged 6 years and older. Duchenne, the most common childhood muscular dystrophy, is caused by dystrophin deficiency. As a histone deacetylase inhibitor, givinostat targets pathogenic processes causing inflammation and muscle degeneration. Givinostat recipients demonstrated less decline in the four-stair climb assessment compared to those receiving placebo (Mercuri et al. 2024). This therapy has received fast track and priority review designation. The most common AEs include diarrhea, abdominal pain, thrombocytopenia, nausea/vomiting, hypertriglyceridemia, and pyrexia. Platelet counts and triglyceride levels should be assessed before starting givinostat treatment.

**Mavorixafor** has been approved as the first therapy for WHIM syndrome (named based on its characteristics of warts, hypogammaglobulinemia, infections, and myelokathexis). WHIM is a rare primary immunodeficiency disease associated with overactive C-X-C chemokine receptor 4 (CXCR4) signaling pathways and the retention of leukocytes in the bone marrow (Hoy 2024). Mavorixafor, an oral selective CXCR4 antagonist, increases the number of circulating mature neutrophils and lymphocytes (Hoy 2024). Clinical trials showed that mavorixafor therapy reduced infection frequency, severity, duration, and antibiotic use (Badolato et al. 2024). This drug has received fast track and priority review designation. Mavorixafor is still under investigation for additional chronic neutropenic conditions. Thrombocytopenia, pityriasis, epistaxis, rhinitis, and dizziness are commonly reported AEs.

In 2024, two drugs were approved for the treatment of Niemann-Pick disease type C (NPC). **Arimoclomol** is prescribed alongside the enzyme inhibitor miglustat for adults and children aged 2 years and older. NPC, a rare lysosomal storage disorder, is caused by mutations in the NPC1 or NPC2 proteins, which regulate cholesterol transport from lysosomes. Arimoclomol modulates heat shock proteins, providing neuroprotective properties against lysosomal stress. It has been shown to reduce the frequency, severity, and duration of infection compared to placebo and is well-tolerated (Benatar et al. 2024). The most frequent AEs include upper respiratory tract infection, diarrhea, and weight loss. Arimoclomol was granted priority review, fast track, and breakthrough therapy designations. The second drug approved, **levacetylleucine**, has been reported to improve neurologic status after 12 weeks of therapy compared to placebo (Bremova-Ertl et al. 2024). This therapy has received fast track and priority review designations. Abdominal pain, dysphagia, upper respiratory tract infections, and vomiting are among the most commonly reported AEs.

**Crinecerfont**, a corticotropin-releasing factor type 1 receptor antagonist, has been approved as a first-in class treatment of classic congenital adrenal hyperplasia in adults and pediatric patients over 4 years of age. Congenital adrenal hyperplasia is a rare genetic disorder associated with insufficient cortisol and excessive androgen production. Crinecerfont is used alongside glucocorticoids to maintain normal androgen levels. It demonstrated superiority over placebo in reducing androstenedione levels and helped reduce glucocorticoid doses (Sarafoglou et al. 2024). Common AEs in adults include fatigue, dizziness, and arthralgia, while in children, headache, abdominal pain, and fatigue are reported. The therapy has received fast track, breakthrough therapy, orphan drug, and priority review designations.

**Olezarsen** is approved as the first therapy for familial chylomicronemia syndrome, a rare genetic disease associated with severe hypertriglyceridemia and acute pancreatitis. Olezarsen is an antisense oligonucleotide designed to target messenger RNA of apolipoprotein C-III and is used as an adjunct to diet. Subcutaneous administration has been shown to reduce plasma triglyceride levels by decreasing hepatic synthesis of apolipoprotein C-III (Stroes et al. 2024). Its most frequent AEs include injection site reactions, decreased platelet count, and arthralgia. This drug has received fast track, breakthrough therapy and priority review designations.

A triple-combination of **vanzacaftor**, **tezacaftor**, and **deutivacaftor** has been approved for the treatment of cystic fibrosis in individuals aged 6 years and older. This once-daily combination of cystic fibrosis transmembrane conductance regulator (CFTR) modulators has demonstrated efficacy and safety by enhancing lung function (measured by percent predicted FEV_1_), respiratory symptoms (evaluated using Cystic Fibrosis Questionnaire), and CFTR function (assessed through sweat chloride levels) (Hoppe et al. 2025; Uluer et al. 2023, Keating et al. 2024). Liver function tests should be performed prior to and monitored during therapy. The most common AE include cough, upper respiratory tract infection, headache, and increased ALT and AST levels.

Additionally, gene therapy has been approved for treating adult and pediatric patients with aromatic L-amino acid decarboxylase (AADC) deficiency. ***Eladocagene exuparvovec-tneq***, a recombinant adeno-associated virus-2-based gene therapy, delivers the human AADC gene. It is administered through bilateral intra-putaminal infusion in a single surgical procedure, with two infusion sites per putamen. The therapy, approved in 2022 in the EU, has been shown to have a favorable safety profile and improvements in cognitive and communication abilities, body weight, hypotonia, and dystonia (Keam 2022, Tai et al. 2022). Dyskinesia was the most frequent AEs, along with insomnia, irritability, and salivary hypersecretion.

## Dermatology

**LetibotulinumtoxinA-wlbg**, a type A botulinum neurotoxin, is approved to temporarily reduce the appearance of glabellar lines. Like other botulinum toxins, it works by inhibiting acetylcholine release and blocking neuromuscular activity, administered via intramuscular injection at different sites. Patients treated with letibotulinumtoxinA showed significant improvements compared to placebo across all measures, including Glabellar Line Scale (GLS), and experienced a reduction in psychological burden (Cox et al. 2024). It should not be administered more frequently than every 3 months. The most common AE is headache.

**Sofpironium** is indicated for the treatment of primary axillary hyperhidrosis. This anticholinergic agent is applied topically at bedtime. Patients using sofpironium showed improvements measured by the Hyperhidrosis Disease Severity Measure-Axillary (HDSM-Ax) score (Kirsch et al. 2020). The most common AEs include dry mouth, vision blurred, application site pain, and erythema. Due to its anticholinergic effects, it is contraindicated in patients with angle-closure glaucoma or benign prostatic hyperplasia (Paik 2020).

**Deuruxolitinib** is indicated to treat severe alopecia areata, an autoimmune disorder affecting hair follicles. It is an oral Janus kinase inhibitor that has been effective in promoting hair regrowth, as evaluated by the Severity of Alopecia Tool score and with patient satisfaction (King et al. 2024). AEs of the therapy include headache, acne, nasopharyngitis, weight gain, increased blood creatine phosphokinase and cholesterol, and anemia. The therapy carries a boxed warning for serious infections, increased cardiovascular, malignancies, and thrombosis.

**Nemolizumab** is approved for the treatment of prurigo nodularis. This monoclonal antibody targets IL-31 receptor alpha, an endogenous inflammatory cytokine. Previously, it was approved for atopic dermatitis in Japan. Nemolizumab is administered via a single-dose prefilled dual chamber pen injected subcutaneously. It has been shown to reduce signs and symptoms of prurigo nodularis (Kwatra et al. 2023). Headache, dermatitis atopic, eczema, and eczema nummula are among the most frequent AEs. Live vaccines should be avoided during treatment. Nemolizumab was granted priority review and breakthrough therapy designations. Another monoclonal antibody targeting interleukin-13, **Lebrikizumab-lbkz**, has been approved to treat moderate-to-severe atopic dermatitis. It is administered subcutaneously to improve symptoms and has a favorable safety profile (Blauvelt et al. 2023, Silverberg et al. 2023). This therapy has received fast track designation. The most frequent AEs include conjunctivitis, injection site reactions, and herpes zoster. Age-appropriate vaccinations should be completed prior to the treatment.

## Diagnostic agents

**Pegulicianine** is a fluorescent imaging agent for the detection of malignant tissue. It is activated by cathepsins and matrix metalloproteases that are highly expressed in the tumor microenvironment (Bou-Samra et al. 2023, Smith et al. 2023). It is administered to the patient prior to lumpectomy and during the operation, the surgical cavity is evaluated for the presence of residual tumor cells by using an imaging system that is sensitive to the fluorescent emission of this agent (Bou-Samra et al. 2023). Pegulicianine has been approved for the intraoperative imaging of cancerous tissue during lumpectomy surgery in adults with breast cancer. This imaging agent has been granted fast track and priority review designation. A multicenter feasibility study revealed that pegulicianine exhibited an acceptable safety profile (Hwang et al. 2022). In a prospective trial, the most common AE was blue chromaturia, which is consistent with the blue coloration of pegulicianine (Smith et al. 2023). The prescribing information of this imaging agent contains a black box warning about hypersensitivity reactions.

**Flurpiridaz F-18** is a radioactive diagnostic pharmaceutical compound that inhibits mitochondrial complex I and has been radiolabeled with fluorine-18 (Yu et al. 2009, Matsumoto 2024). In a phase 3 study, positron emission tomography myocardial perfusion imaging with flurpiridaz F-18 for the detection of coronary artery disease was found to be superior to technetium-99m-labeled single photon emission computed tomography imaging, particularly in female subjects and obese patients (Maddahi et al. 2023). Flurpiridaz F-18 has been approved for positron emission tomography myocardial perfusion imaging under treadmill or pharmacologic stress testing to assess myocardial ischemia and infarction in adults. The most common AEs were respiratory distress, pain, cardiovascular disorders, tiredness, flushing, nausea, and dizziness, according to prescription information.

**Iomeprol** is a non-ionic, monomeric, radiographic iodinated contrast agent with reduced toxicity, osmolality and viscosity, and elevated water-soluble properties (Dooley and Jarvis 2000, Katayama et al. 2001b). Iomeprol solutions have the advantage of chemical stability, which does not require adding chelating agents (Dooley and Jarvis 2000). It has received approval for utilization in intra-arterial and intravenous procedures, including arteriography, ventriculography, radiographic assessment of cardiac chambers and arteries, and computed tomography. Following administration, iomeprol induces opacification in the body areas where the contrast medium is distributed, thereby facilitating radiographic imaging (Katayama et al. 2001a). The prescribing information includes a black box warning that intrathecal administration may cause fatal AEs. The most common AEs were elevated body temperature, gastrointestinal disorders, and pain.

## General trends and conclusions

As in previous years (Kayki-Mutlu and Michel 2021, Kayki-Mutlu et al. 2022, Kayki-Mutlu et al. 2023, Kayki-Mutlu et al. 2024), oncology had the greatest share of newly approved drugs with 17 approvals representing 34% of all approvals. Interestingly, we observed a trend for an increasing number of approvals in oncology that are based on specific tumor genotypes. Hematology/immunology was the second largest group of approvals based on nine approvals, i.e., 18% of all. Genetic disorders (8; 16%) were similarly prominent. While the combined field of cardiovascular and respiratory approvals (6; 12%) came in fourth, all other therapeutic areas had four or less approvals (i.e., <10%). Following our previous reviews (Kayki-Mutlu and Michel 2021, Kayki-Mutlu et al. 2022, Kayki-Mutlu et al. 2023, Kayki-Mutlu et al. 2024), others have followed this example applying slightly different approaches but generally came to the same overall conclusions. For instance, they applied somewhat different definitions of novelty (first-in-class status) (Papapetropoulos et al. 2024).

**Fig. 1:**
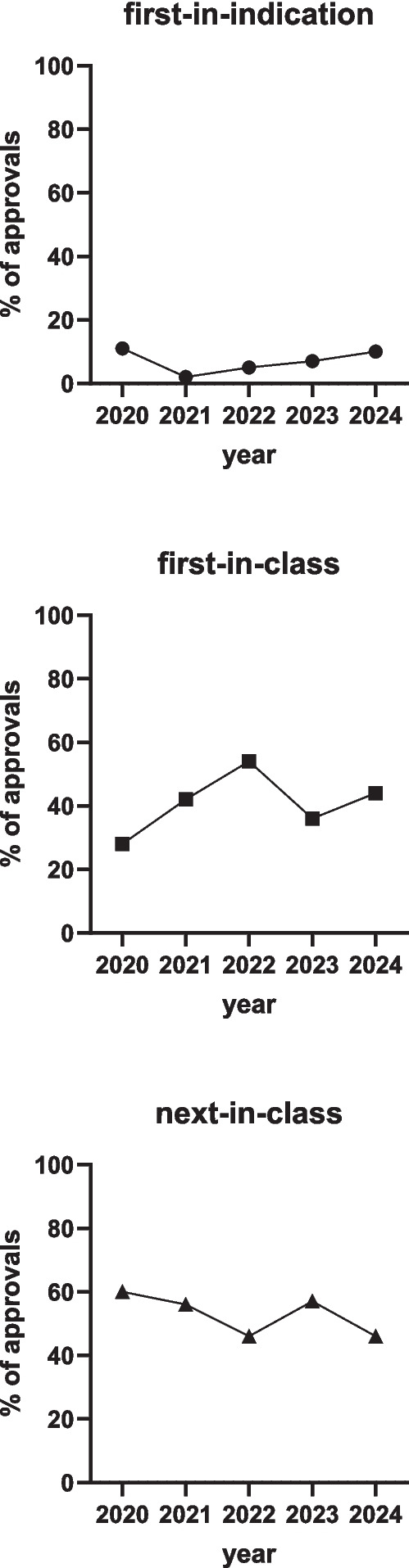
Degree of innovation in drugs approved in 2020–2024

Looking back at the past 5 years for which we performed this type of analysis, we can now look at trends over time. For this we focus on three aspects of new approvals. Firstly, we look at the degree of innovation which we define as first-in-indication (no other drug available for this condition), first-in-class (no prior drug using this molecular mechanism of action), and next-in-class. First-in-indication approvals ranged between 2 and 11% of all approvals in 2020–2024 (Fig. 1). While this largely affects orphan indications and thus not many patients, those afflicted by such a condition will certainly profit from any medical treatment option becoming available, implying that reliance on off-label use of medications or, in a worst case, no treatment at all is over. First-in-class approvals ranged between 28 and 54% in 2020–2024, with no clear trend over time. First-in-indication and first-in-class approvals combined testify to the innovation power of the pharmaceutical industry. Next-in-class approvals (60% of all approvals in 2020 and 41% in 2024) exhibited some fluctuations over time, but overall this class appears declining. Of note, a next-in-class approval does not necessarily imply lack of innovation as compounds from previously introduced drug classes still may represent clinically relevant progress by exhibiting greater efficacy, improved tolerability or, specifically in oncology, efficacy in disease types that have grown resistant to previously introduced members of a drug class.

**Fig. 2:**
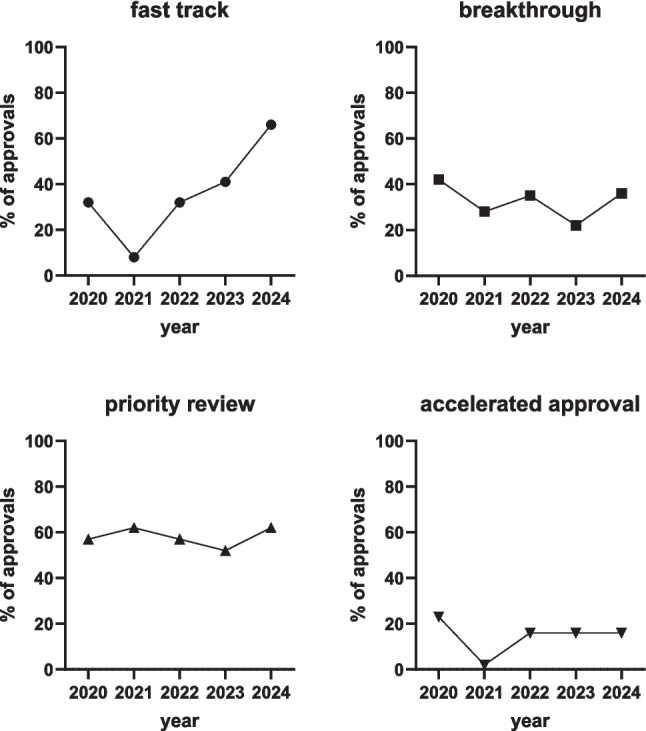
Approval paths for drugs approved in 2020–2024

Secondly, we looked at the frequency of the FDA applying specific forms of approval. Several years ago, the FDA introduced new approval processes named priority review, breakthrough therapy, fast track, and accelerated approval. In this regard, priority review means that the FDA aims to evaluate these drugs within 6 months compared to a standard review that requires 10 months based on a treatment being considered to provide a marked improvement in the therapy, diagnosis, or prevention of severe disorders (U.S. Food and Drug Administration 2018d). The definition of breakthrough treatment is partly overlapping as it also implies an accelerated evaluation process for treatments to treat severe conditions because the new treatment is considered to exhibit more clinically relevant outcomes than current treatments based on preliminary data (U.S. Food and Drug Administration 2018b). The fast-track designation implies that the overall evaluation process is accelerated to make critical drugs available to patients as early as possible, for instance, for serious conditions such as Alzheimer’s, heart failure, and cancer, when existing treatments are considered insufficient (U.S. Food and Drug Administration 2018c). Finally, accelerated approvals are granted based on a surrogate endpoint from which clinical benefit can be predicted for severe conditions that need innovative medical treatments (U.S. Food and Drug Administration 2018a). Accelerated approvals imply that post-approval clinical studies are mandatory. If those post-approval studies show a considerably smaller benefit and/or unexpected intensity or frequency of AEs, i.e., an overall major shift in the benefit/risk ratio, drugs given accelerated approval may be withdrawn in the light of new data. An example of such withdrawal was the oncology medication melphalan flufenamide (Olivier and Prasad 2022). Looking at the trend of the past 5 years, we see that the fraction of newly approved treatments receiving breakthrough (22–42%), priority review (52–62%), or accelerated approval (2–23%) remained stable (Fig. 2). However, we observed a trend for increasing use of the fast-track approval pathway; this path reached more than 50% of all approvals in 2024 (Fig. 2).

**Fig. 3:**
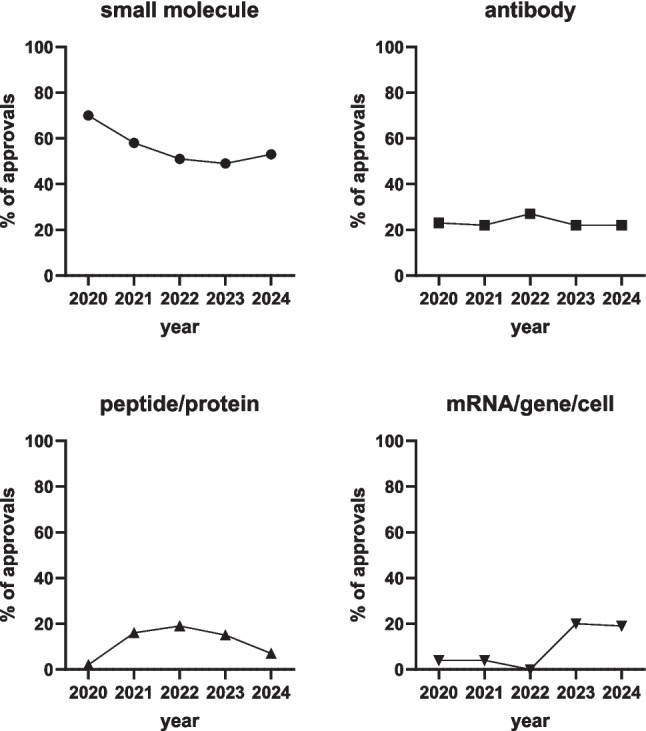
Drug types of approvals in 2020–2024

Finally, we look at trends for drug classes. Small molecules historically were the bedrock of pharmacotherapy, partly because no other drug types were available. The past 5 years show a decreasing trend in approvals of small molecules (from 70% in 2020 to about 50% in 2022–2024; Fig. 3). While the approval of antibodies (22–27%) and of peptides/proteins (2–19%) fails to show a clear trend, approvals of mRNA-based treatments, gene and cell therapy shows a major increase reaching about 20% of all approvals in the last 2 years (Fig. 3).

## Data Availability

All source data for this work (or generated in this study) are available upon reasonable request.
